# Book Reviews

**Published:** 1807-11

**Authors:** 


### CRITICAL ANALYSIS
### OF THE
### RECENT PUBLICATIONS
### ON THE
### DIFFERENT BRANCHES OF PHYSIC, SURGERY, AND MEDICAL PHILOSOPHY.

---

*An Essay on the Nature of Fever, being an Attempt to ascertain the Principles of its Treatment.* By A. PHILIPS WILSON, M. D. F. R. S. Ed. and Fellow of the Royal College of Physicans of Edinburgh, &c. 8vo. London.

A SHORT Advertisement precedes the Introduction, in which the author defines the meaning which he attaches to the term *proximate cause.* After observing that different writers have affixed different meanings to this expression; the following, we conceive, we are to consider as *his* definition.

"The proximate cause has been defined, that state of the body which, when present, causes; when removed, removes; and when changed, changes the disease. When we have ascertained any cause to which this definition applies, and pointed out the means of removing it, we have done all that is necessary. These, therefore, ought to be our objects in treating of the nature of disease. It signifies not what minute motions of the system produced this cause, or by what minute motions the means which remove it, operate.

"The word proximate cause is objectionable; it by no means expresses the idea which the best writers affix to it, and has led to fanciful theories respecting these minute motions of the system, which are doubtless the more immediate, and therefore, strictly speaking, the proximate cause of all diseases, but which we cannot trace, and which, if we could trace, it is probable we could not regulate."

We confess ourselves no better satisfied with the above, than with any former definition. For if "that state of the body which, when present, causes; when removed, removes; and when changed, changes, the disease," means any thing more than the fever itself, it must, we conceive, refer to some *cause*, which will for ever evade our senses, and which, if we cannot trace, we cannot hope to regulate.

An Introduction follows, in which the order of the work is announced, as well as that succession of facts and reasoning by which the author has gradually matured his opinions. In doing this, the theories of others are slightly glanced at, and the position of Dr. Cullen is much insisted on, that a too frequent reference to the *vis medicatrix naturæ*, has greatly retarded the inquiry into the proximate causes of fever. The

The work itself begins by a short Review of the opinions of others respecting the proximate cause of fever. We shall omit all that is said before the time of Dr. Cullen, as we conceive the humoral pathology has now scarcely a surviving disciple. Our principal object being directed to Dr. Wilson's opinions, we shall only transcribe the concluding summary of the Cullenian doctrines, with some of our author's remarks thereon.

"Upon the whole," says Cullen, "our doctrine of fever is explicitly this: The remote causes are certain sedative powers applied to the nervous system, which diminishing the energy of the brain, thereby produce a debility in the whole of the functions, and particularly in the action of the extreme vessels. Such, however is, at the same time, the nature of the animal œconomy, that this debility proves an indirect stimulus to the sanguiferous system, whence, by the intervention of the cold stage and spasm connected with it, the action of the heart and larger arteries is increased, and continues so till it has had the effect of restoring the energy of the brain, of extending this energy to the extreme vessels, of restoring therefore their action, and thereby especially overcoming the spasm affecting them; upon the removing of which, the excretion of sweat, and other marks of the relaxation of excretories take place."

"From the parts of the preceding quotation, which are printed in Italic letters, it is evident that Dr. Cullen regarded his doctrine of fever as little more than an hypothesis, calculated to give arrangement to detached facts, which, without some system, readily slip from the memory.

"He believed, indeed, and it must be admitted, that it was founded on a better principle than that of any of his predecessors, except Hoffman; and he perceived that it was more simple and consistent than the doctrines of this writer; but he looked forward to a more advanced state of science, in which his system would suffer a change similar to that which he had effected en Hoffman's. All this he expresses fully in the Preface to his *First Lines*."

"Upon this general plan," he observes, "I have endeavoured to form a system of physic that should comprehend the whole of the facts relating to the science, and that will, I hope, collect and arrange them in better order than has been done before, as well as point out, in particular, those which are still wanting to establish general principles. This which I have attempted may, like other systems, hereafter suffer a change; but I am confident that we are at present in a better train of investigation than physicians were in before the time of Dr. Hoffman."

"But setting aside what Dr. Cullen says of his doctrine, if we examine the doctrine itself, we shall find that it is wholly constructed on a hypothetical basis, on the supposed operations of the vis medicatrix naturæ, respecting which we have, in the Inttroduction to this Essay, given Dr. Cullen's opinion.

"How

"How the debility of the nervous, proves an indirect stimulus to the sanguiferous, system; how this stimulus acts in exciting the cold stage and spasm; how, through the intervention of these, the action of the heart and larger arteries is increased; is only explained by reference to the operation of the vis medicatrix, that is, is not explained at all; and yet it appears, even at first view, that on these the whole system rests.

"Shall we suppose Dr. Cullen, after declaring 'that where-ever the vis medicatrix is admitted into medical systems it throws an obscurity on them,' so inconsistent as to offer as a true system one wholly and avowedly founded on the supposed operations of this agent!"

Dr. Brown's system is detailed more at large, and to do justice to the author of the work before us, that detail is more candid, as well as perspicuous, than any we have met with among the numerous adversaries of that unfounded system. It shews with much perspicuity, that Brown felt it impossible, if not dangerous to his opinions, to give any where a regular statement of his doctrine. That he proved, what no one would dispute, that when a man is tired he must rest; that certain substances proved a stimuli to an organization, which, without such stimuli, would cease to move, and that when such stimuli could no longer excite action, that excitability was extinguished, and the organization could no longer be maintained.

The principal objection made by Dr. Wilson to Brown's Theory is, that no distinction is made between the series of action in those organs which he calls vital, whose functions are constantly necessary for the maintainence of life, and others which he calls animal organs, whose actions are only occasional for the comfort of the animal, and for enabling him to supply those deficiences in the system, which the constant operation of the vital organs induce. Other objections are made to the theory, as confounding the state of excitement in health with that under disease: and also, that no distinction is made between the various kinds of stimuli, or agents, their effects, and the means of removing them. Thus direct debility being that state in which excitability is accumulated, must, under some circumstances, be a state of health, and the various shades of accumulation tending to disease can never be ascertained. The passions are agents of a peculiar kind, the effects of which cannot be removed, but by the application of similar agents, whether they are called stimuli or not.

"*The hypothesis of direct debility* supposes, that the abstraction of any one of these agents renders the system more sensible to every agent, and that the effect of all is, at all times, excitement.

"*The facts* are, that the abstraction of any one of these agents only renders the body more sensible to the action of that agent; and the effect of that agent is not always excitement, but either excitement or atony, according to the degree in which it is applied, plied, and the state of the body at the time of its application, that is, according to the change it induces.

" If the change is moderate, it proves a stimulus; and, within a certain range, the greater the change the greater is the excitement. Beyond this, as we have seen in the instances of opium and distilled spirits, it occasions debility; and, when excessive, death.

" When the change induced is consistent with the health of the parts on which the agent acts, excitement is the consequence; but when the change is sufficient to derange the mechanism of the living solid, if I may use the expression, its immediate effects are debility or death. Nor is this more remarkable of the agents which are directly applied to the living solid, than of those whose first impression is on the mind. The passions, within a certain degree of intensity, act as stimuli; beyond this they debilitate, and even extinguish life, without previous excitement.

" The degree of exhaustion which follows the operation of any agent is always proportioned to the excitement it occasions; but the degree of atony which a greater quantity of the same agent produces, bears no proportion to its exciting power. Thus tobacco will not occasion the same degree of excitement which opium or distilled spirits do, but it is better fitted to produce atony.

" Of those agents whose first impression is on the mind, some, grief, fear, disgust, are ill calculated to excite, although, when present only in a small degree, they act as stimuli; but they are chiefly calculated to produce atony; others, love and joy, on the contrary, produce much excitement, and only occasion atony when in excess.

" With respect to what Dr. Brown says of the depressing passions, as it makes a part of his hypothesis of direct debility, it must fall with that hypothesis; unless we allow that grief, fear, &c. occasion an accumulation of excitability, there is nothing which Dr. Brown says on this subject that can be admitted. According to a law of the animal economy, I have just had occason to mention, those under the operation of grief are rendered more sensible to joy, and those under the operation of fear to confidence; but they are rendered less sensible to the operation of every other agent. His assertion that grief is only a less degree of joy, and fear nothing more than a diminution of confidence, is quite gratuitous. He might with equal reaon assert, that confidence is a dimiuntion of fear, and joy a less degree of grief. The one set of passions are as positive agents as the other: and if the one tend more to excite, and the other to depress, it is only what is true of agents of every other species."

Dr. Brown, it is added, terms those *diseases* sthenic, in which a greater degree of excitement exists than in health. But such an excitement, if really diseased, is followed by atony. For, if mere exhaustion follows, which may be relieved by sleep, such a state state cannot be called diseased. Or, in other words, if excitement produces exhaustion only in the animal powers, which may be readily restored ; we are not to confound such a state with a morbid excitement, which debilitates the vital, as well as the animal functions.

"Upon the whole," adds our author, "the following, as far as I am capable of judging, are the facts which Dr. Brown overlooked in forming the great outlines of his hypothesis.

"There is no accumulation of excitability beyond that which constitutes a state of the most perfect vigour. There is no exhaustion of excitability, in the sense in which Dr. Brown uses the term, beyond that which constitutes the most perfect sleep, and both are equally states of health.

"Every agent is capable of producing either excitement or atony, according to the degree in which it is applied.

"In health, the natural agents applied in the usual degree, viz. a certain temperature, a certain quantity of exercise, &c. always occasion that kind of excitement which is followed by exhaustion.

"In general disease, that is, in fever, which is the only general disease properly so called, the state of the excitability is so changed, that the same agents do not produce a greater or less degree of the same effects they produce in health, as Dr. Brown supposes ; but either atony, or that kind of excitement which is followed by atony.

"It must appear, I think, to every one who attentively considers the hypothesis of Dr. Brown, that its author, in speaking of diseases, has constantly in view the healthy state of the animal body ; and attempts, in vain, to apply the laws which regulate the excitability of certain parts of the system in health, to explain the phenomena of disease."

This leads our author to his own theory of the proximate cause of fever. He begins, by dividing the functions of the human body, as we observed when reviewing Dr. Clutterbuck's work, into the vital and animal.

"The one set of these functions is evidently designed for the support of the other. The end of animal life is to feel and communicate enjoyment : The animal functions, or, to speak more accurately, their immediate organs (which, to save repetition, I shall call *animal organs*, in contradistinction to the organs properly called *vital)*, are the immediate instruments of both ; and they are maintained by the vital organs, whose office is to form and nourish them, and, when their vigour is exhausted by the impressions which excite them, so that they cease to act, to refit them for receiving and answering those impressions.

"It is evident, therefore, that when the vital organs are debilitated, the animal organs necessarily partake of the debility. It is equally evident why the converse of this is not true, the nervous system may be debilitated without affecting the powers of of circulation. Thus when any cause impedes the action of the heart, the brain immediately partakes of the disorder: but even in apoplexy the pulse is generally strong and good, and only becomes slower and more languid in proportion as the respiration, which is performed by muscles that depend for their vigour on the influence of the brain, becomes so.

"Thus it is that in diseases properly termed nervous, the pulse is generally good, however great the languor and depression of strength may be; nay I have even found, from experiments made for the purpose of ascertaining this point, that the total destruction of the nervous system produces no immediate effects on the action of the heart, nor in any other way affects its motion than necessarily happens in consequence of the interruption of respiration."

Dr. Wilson then proceeds to shew, that the great error of Brown, was in not perceiving the terms excitement aad exhaustion, could only apply to the animal functions, and are merely expressions of the healthy state of vigilance and sleep. That while the vital organs retain their vigour, the animal organs can feel no debility which can be considered as morbid. But, that should the vital organs be debilitated from any cause, instead of mere exhaustion, atony follows, which cannot be restored like exhaustion in the animal functions. To restore the vital powers, a stimulus stronger than the natural stimulus must be applied; the system possessing no organs, "by which the vigour of those of assimilation, that is, of the vital organs can be restored."

"The application of a preternatural stimulus to them, is the unavoidable consequence of their debility: for the secreting organs no longer performing their functions, the more irritating and noxious parts of the blood, which ought to be expelled from the body, are retained, and soon excite the heart and blood vessels to an action as powerful as the healthy action, often more so."

The author proceeds to remark, that all debilitating causes applied to the vital organs are felt most at those parts which have least power, that is, at the greatest distance from the heart and arteries. Hence, the various secrections fail. These may be restored, if the fore-mentioned stimulus sufficiently answers the action of the heart and arteries. If not, that the debility will gradually extend to the heart and arteries themselves, and death follow.

"It appears, then," concludes our author, "that when a debilitating cause is applied to the vital system, the extreme parts of this system lose their tone; that, in consequence of this, secretion being impeded, a preternatural stimulus is applied to the heart and larger vessels, which, by exciting them, tends to restore tone to the capilliaries, in the same way that an increased action of the larger vessels of an inflamed part tends to restore tone to the capillaries of that part. On this principle, I believe, the whole phenomena of fever may be explained."

When

When we read this passage, it affords ideas so precisely similar to Dr. Cullen, that, we confess ourselves unable to see any difference, excepting that the Professor acknowledges himself dissatisfied with his theory; whilst Dr. Wilson promises us "to establish his position by reviewing the three heads of the Symptoms, Causes, and Cure of Fever."

Under the first article, we find the enumeration of almost every symptom; and, like most other systematic writers, the author feels no difficulty in accounting for all, according to his favourite hypothesis. He goes indeed a little out of his way, to show that Cullen was mistaken in imputing any of these symptoms to a putrescency of the fluids, induced by marsh or human effluvia becoming putrefactive ferments. These opinions, not only have not survived the author, but died before him. But we feel no greater satisfaction from Dr. Wilson's remarks on the putrefaction of the contents of the stomach and alimentary canal by stagnation, or of the stagnation and putrefaction of the natural moisture on the surface, by the failure of the secretion and absorption at those parts. This putrefaction, as it is called, and this cadaverous smell, we impute to an alteration in the secretions themselves, and not to any particular change after the process of secretion.

The remote causes of fever are next enumerated; and we are assured by the author, that whatever they may be, whether cold, fatigue, or contagion, the operation of each, is to induce debility. These are subjects we shall not dispute with Dr. Wilson.

Under the last article of Treatment, little novelty could be expected. Some judicious hints occur, besides the common routine practice; and if we are not satisfied with the author's mode of reasoning upon any, we strongly suspect that our readers will excuse us, when we decline entering into an argumentative detail on the subject.

Such is the nature of the work before us. It is particularly unfortunate for the author, that Dr. Clutterbuck should have so immediately preceded him. Though we are not perfectly satisfied with either, yet, it is impossible not to compare the solid reasoning of the one with the unfounded theories of the other.

---

### *The Edinburgh Medical and Surgical Journal, No. XII.*

Article 1.—*A Letter to Baron Jacobi Kloëst, Envoy Extraordinary, and Minister Plenipotentiary from the King of Prussia to the Court of Great Britain, from* Gilbert Blane, Esq. F. R. S. S. London and Edinburgh, Corresponding Member of the I. A. S. of St. Petersburgh, and Physician to His Royal Highness the Prince of Wales, *respecting the Nature and Properties of the Yellow Fever.*

His Prussian Majesty became so much alarmed lest the Yellow Fever should find its way into his dominions, that he offered a prize prize medal to the author of the best treatise on the subject. The words of the Conspectus, relative to this question are so remarkable, that we cannot help copying them for the instruction of our readers, that is, that they may avoid falling into similar difficulties.

" *Conspectus quæstionis, a Collegio supremo medico Berolinensi, modum contagii febris sic dictae flavae spectantis, propositae.*

" Febrem flavam illis jure adnumerari morbis, quibus peculiare est ab ægris ad sanos ope contagii transferri, experientia sat superque edocti fuimus.

" Patet inde, contagiosum virus huic morbo proprium existere, quod ex illo gignitur et causam propagationis illius continet.

" Quo vero modo contagium proseminetur nobis attamen hactenus non satis innotuit, sed ita latet, ut in dubio hæreamus; an ægrorum contactus solus contagionem febris flavæ producat? vel an virus per atmospheram transferatur et tali modo sanos contaminare valeat? vel tandem: an miasma febris flavæ, pestis miasmatis ad instar, corporibus omnigenis adhæreat et illos ita inficiat, ut eorum contrectatio ad producendum morbum istum contagiosum, sufficiat?"

Thus it seems, the College of Berlin is satisfied that the Yellow Fever is a contagion which may be transferred from the sick to the healthy, and the only doubt is, whether contact of the sick is necessary to produce this effect, or whether the virus may be transferred through the atmosphere retaining its contagious properties, or whether the miasma, like that of the plague, may adhere to bodies of every kind, and so affect them, that their contact may be sufficient to induce the disease.

Had this learned body maturely considered the nature of their position and consequent question, they must have recollected that we know of no contagions requiring contact with the sick, to produce their effect, excepting such as induce their diseased action on the part touched. It would, therefore, have been desirable to illustrate their meaning, by exemplifying the venereal, or some other poison producing its effect in this way. When it is said of the plague, as it has been by some people, that it is only contagious by contact, and when it is considered at the same time, that we can find no visible change in the part thus touched; that contact is often not followed by any consequent disease, and that those who are not exposed to contact, are sometimes affected; the only inference we can draw, is, that if the disease be contagious, the laws of that contagion is not yet understood, and therefore, that every person gives his surmise according to the individual facts which have passed before him. Having said thus much on the question, let us now attend to the manner in which it is answered. In the paper before us, Dr. Blane begins by observing, that his various duties and occupations will not admit his drawing up an answer agreeably to the form prescribed, but as the disease in question has come largely under his notice, and as he has been led to pay particular attention attention to it at home and abroad, he hopes to relieve his Prussian Majesty from his anxieties relative to the public health from this *epidemic*.

It is next observed, that the disease has never appeared, excepting in tropical climates, or in those seasons when the temperature has been for some time equal to the tropical heat. This fact, Doctor Blane seems particularly desirous of "communicating to the Prussian Government, in order to remove any groundless fears with regard to the dangers to be apprehended to the states of his Majesty from this *infectious* disorder. In this respect, it is said to "differ from Small-pox and other *specific contagions*, but is very analogous to the plague, for it is perfectly ascertained, that the prevalence of this latter *epidemic* is confined to a particular range of atmospheric heat, both in regard to climate and season."

Another property of Yellow Fever we are told, is, "that it is never known to prevail but where the effluvia of the *living* human body existed in a certain degree of concentration." Dr. B. has remarked, that the British armaments sent to the West Indies, have suffered most when numbers were confined to a spot, and "in his work on the diseases of seamen, that this *epidemic* was most apt to appear in the crews of crowded ships, bringing *febrile infection* from England or America."

It is next stated, as a very striking property, that the disease has never been known to spread into the country among villages or single houses. Here again we are shown the resemblance of the Yellow Fever and plague, the infection of the latter having a "great predilection to the crowded and ill aired parts of the town, on its first breaking out; but in its after progress, it spares no quarter of the town, nor even the smallest villages in the career of its devastation."

Our author next attends to the opinions of those people, who, from the circumstances above related, have been led to suppose that the Yellow Fever is not infectious, and to others who have entertained the same "paradoxical opinion of the plague itself."

"The infection of both, it is urged, may be aptly compared to the seeds of vegetables, or the eggs of animals, which require a nice concurrence of certain degrees of heat, moisture, rest, nutriment, &c. to animate them. Infectious matter has, by a very appropriate metaphor, been termed the *seeds* of the disease; and, by a similar propriety of expression, it has been said, that a certain *nidus* is necessary to give it effect. The *nidus* in plague is a certain range of atmospheric heat, which is a requisite *sine qua non;* and a certain concentration and corruption of animal *effluvia*, which is not so indispensable, but which greatly facilitates the catching and propagation of it. The *nidus* of the Yellow Fever is also a given range of atmospheric heat, and a certain concentration and corruption of animal *effluvia*, both equally indispensable. It would be too tedious to enumerate the various proofs, derived from my own own observation, and the testimony of others, in proof of the *infectious* nature of Yellow Fever, I shall content myself with citing one, taken from my letter to Mr. King, Minister of the States of America to this Court, who applied to me, on this subject, in the year 1798. On the 16th of May, 1795, the Thetis and Hussar frigates captured two French armed ships from Guadaloupe, on the coast of America. One of these had on board some men ill of the Yellow Fever; and, out of fourteen hands, sent from the Hussar to navigate and take care of her, nine died of this fever before she reached Halifax, on the 28th of the same month, and the five survivors were sent to the hospital, sick of the same distemper. Part of the prisoners were sent on board of the Hussar; and, though care was taken to select those seemingly in perfect health, the disease spread rapidly in that ship, so that near one third of the whole crew was more or less affected by it.

"It is now, therefore, manifest, that different species of infection are regulated by different laws. The small-pox propagates itself in all seasons, climates, and situations. The plague cannot exert its virulence, but under a range of heat above the 60th, and below the 80th degree of Fahrenheit's thermometer. The Yellow Fever never spreads but under still higher degrees of heat, and in situations where the air is contaminated with human *effluvia*: and the practical inference is, that there is no hazard of its becoming epidemic in the north of Europe."

A very short sketch follows of the first appearance and subsequent progress of this disease in the West Indies, in America, and in Europe; in all which, historical proof is produced, that the disease has been constantly imported. After which the error of those authors is noticed, who allege "that the Yellow Fever is produced by the same marshy and putrid exhalations which produce the intermittent and remittent fevers, and that it is only a variety of the latter. But the remitting fevers differ from it in some essential symptoms; and the Yellow Fever has been known to arise, both in ships and on shore, where men were not exposed to any corrupted exhalations of the soil, nor to pernicious vapours of any kind, except such as proceed from the contaminated effluvia of the living human body.

"In short, all the difficulties respecting the contagious nature of this disorder are got over, by considering that the poison by which it is propagated, in common with other species of infectious matter, takes effect in consequence of a delicate and casual concurrence of incidents, compounded of internal predisposition, and external circumstances; and if this were not the case, every infectious disorder would spread to the whole of mankind, and the more malignant epidemics would extinguish the human race."

The inferences drawn from all the facts witnessed by Dr. Blane, or which he could collect from others, is, that the North of Europe is not susceptible of the disease; that in the South of Europe and North America, rigid precautionary steps should be taken,

 not not against the introduction of merchandise, but persons and their foul linen or other clothes, because though there has been an uninterrupted intercourse between the American towns and the West Indies, as long as the fever has been known in the latter, yet no proofs have occurred of the introduction of the disease, but by the sick or their clothes. Another preventive means of the utmost importance consists in paying the strictest attention to ventilation and the cleanliness of towns, particularly by the introduction of common sewers."

Such are the opinions of, and thus are they expressed by the author of "Observations on the Diseases of Seamen," the physician to the British fleet, &c. and we cannot help remarking how fortunate it is that men may practice well and reason very badly. It is impossible to doubt the integrity, industry, or practical judgment of Dr. Blane; and if we venture to say his reasoning is unsatisfactory, if not unintelligible, he may at least be satisfied with being placed on the same footing with the illustrious Sydenham!

The first thing that must strike every reader, is, that the same disease is indifferently called *epidemic*, *contagious*, and *infectious*, and that we are no where informed what is the precise meaning affixed by the author to any of these expressions. We find, indeed, a sort of distinction between *specific contagions* and the yellow fever. Among the former, the small-pox only is *specified.* Now let us ask what is meant by specific. If it is only that such contagion produces its own species, we must remark that Dr. Blane attempts to confine the Yellow Fever to certain laws, by which this *species* of *infection* may be detected in various parts of the world through which he traces it. "The most distinguishing symptoms says he, of the Yellow Fever, are, a yellowness of the skin, the vomiting of a dark coloured fluid, its generally proving fatal from the third to the seventh day, and marks of inflammation on the stomach upon inspection after death."

"It is, says our author, manifest that different *species of infection* are regulated by different laws. The small-pox propagates itself at all seasons, climates, and situations." The plague and Yellow Fever only under certain circumstances. By this, all the *infections* seem referable to some one species; but if we are to make a distinction between general and specific, according to certain laws, the small-pox which propagates itself in all seasons, climates, and situations, seems of all others the least entitled to be restrained by the terms *specific.* We wish our readers to understand that they are not to consider these remarks as etymological, or that we have any intention to object to any terms or qualifications of them by which Dr. Blane may chuse to illustrate his meaning. Our objection is, that we cannot by the closest attention to his paper, derive those benefits from it which we might have done, had he explained all his terms, or avoided applying the same terms to qualities which he wishes to separate.

Let us now attend to the laws which Dr. Blane conceives established.

blished. The first is, that Yellow Fever is unknown, excepting in tropical heat. This we believe is pretty universally admitted.—Besides this, using a metaphor from vegetation, he tells us that "the *nidus* of the Yellow Fever is a given range of atmospheric heat, a certain concentration and corruption of animal *effluvia*, both equally indispensible." Before this, we are told that this fever has never been known to prevail but where the *effluvia* of the *living human* body existed in a certain degree of concentration. These last words admit of no question as to their import. The former terms are therefore too general, and the word *corruption* is improper, as it might lead the reader to imply the putrefaction of *dead animal matter of any kind.* But as we have just remarked, the author's terms are precise as to the cause of Yellow Fever, namely a high temperature and the concentration of living human effluvia. "The British armaments, he adds, sent to the West Indies, have always suffered most when most crowded; the *epidemic* is most apt to appear in the crews of crowded ships, bringing febrile *infection* from England or America," "is more common in ships of the line, especially, if not clean or well aired, than in frigates or other smaller vessels." After this, it is thought sufficient to cite one proof among the many which it would be too tedious to enumerate, that the disease is infectious: this proof is, that a fever on board a French prize proved infectious to the British captors; and in another place the Yellow Fever has been known to arise, both in ships and on shore, where men were not exposed to pernicious vapours of any kind, excepting such as proceed from the contaminated effluvia of the *living* human body."

From all this, it is evident that Dr. Blane has so far lost sight of his subject as to give an account of the jail, ship, or hospital fever, as it affects the human body, in warm climates, instead of the Yellow Fever of the tropics, and of the hot season in North America. It may be urged, and we are ready to admit that the typhus fever of England has different symptoms from those which Dr. Blane describes as characterestic of Yellow Fever. But the great object of the paper is to show the cause of, and to instruct the world in the means of, avoiding Yellow Fever. If then these causes are imputable only to heat and the effluvia of the *living human* body, to what purpose are we to attend so strictly "*to the universal introduction of common sewers.*" In which of the North American towns are the inhabitants so crowded, as to induce that concentration of *living human* effluvia, which Dr. Blane found so destructive on board ill ventilated ships. Philadelphia, which has most to deplore, has streets more spacious than any other town in the world. But Philadelphia was once in want of sewers, and its buildings out ran the necessary supply of water. In consequence of this, her streets were filled with *corrupted dead* animal matter, the offals of slaughter houses, and even the refuse of the kitchens of its well fed inhabitants. These, and not living human effluvia, were considered the cause of the fever, when the atmospheric temperature hastened putrefac-

 tion, tion, and long continued calms aided the accumulation of the evolved gasses. That the ship or camp fever in tropical climates should assume many of the symptoms of a fever peculiar to those regions is not to be wondered at, when we consider the violence of the common remittent, which is often imputed to no other causes than such as in colder countries produce common ague. But whether thus much is admitted or not, we shall not undertake to decide; yet we cannot help expressing our wish, that in so important a question, Dr. Blane had given us some better reasons for suscepting the contagious nature of the Yellow Fever of America, than his history of a fever, communicated by a French prize.

What surprises us still more, is the accuracy assumed in tracing the introduction of the disease to the sick and the clothes about them. "Though, says Dr. B. the disease has prevailed in the West Indies for 100 years, and though there has been a frequent and uninterrupted intercourse of trade between these Islands and North America, we have seen that for 30 years together, Philadelphia and other towns in that country have been free from it." Is it possible to conceive, that during this time, these towns having "a frequent and uninterrupted intercourse of trade with the West Indies, should never have received the sick or their foul linen, or other clothes during their hot season! But if it has invariably happened, that the fever has only occurred during a high temperature and long calms; shall we boldly undertake to ascertain, contrary to the observations of some of the best informed practitioners of America, that the presence of a subject, or of a fomes, is necessary to excite such a disease?

We have dwelt so long on this paper, on account of the celebrity of its author, as well as the importance of the subject. We sincerely wish he would peruse what has been written by others, who, though less acquainted with "the diseases of seamen" have much larger opportunities of tracing the Yellow Fever. Dr. Rush's opinions, though once violently opposed, are gradually gaining ground among his countrymen, and their improvements in cleanliness, seem likely to supersede the imposition of vexatious and unnecessary quarantines.

Article 2.—*Further Observations on the Egyptian Ophthalmia, as it affected the 2d Battalion of the 52d Regiment. In a Letter to* George Peach, Esq. Surgeon to the 2d Battalion, 52d Regiment, to James M'Gregor, M. D. Fellow of the Royal College of Physicians, Edinburgh, and Deputy Inspector General of Hospitals, Portsmouth.

In the first part of this paper, the author shows the great advantages which attended copious venæsection in the manner recommended by Mr. Knight. One man was bled to the amount of 77½ ounces. By an attention to this bold practice in the early stage, the disease was entirely subdued. In some varieties of the complaint, advantage was found in destroying the small arteries by lunar caustic; some useful practical remarks follow, on the means of preventing the extension of the disease. Article

Article 3.—*A Case of Scirrhus Ulceration of the Pharynx and Œsophagus.* By GEORGE KITSON, Member of the Royal College of Surgeons, London.

THIS is one of those melancholy cases in which physic has hitherto made scarcely any progress, excepting to teach us how little can be done. What is most surprising is, that though the patient from inability to swallow, had for a considerable time been confined to little more than a tea-cup full of food in the course of the day, yet she retained strength sufficient to accomplish her customary duties as a servant, and less than three weeks before her death, walked eight miles to see her friends, returning on the following day.

Article 4.—*Case of Luxation of the Femur from an apparently slight Cause.* By C. S.

Article 5.—*Case of Popliteal Aneurism.* By JAMES DAWSON, Esq. Surgeon, Liverpool.

IN performing this operation, Mr. Dawson availed himself of the high operation, as proposed by Mr. Hunter, of the crooked needles in the artery, as proposed by Mr. H. Cline, and of the thin ligatures to divide the inner coat of the artery, as proposed by Mr. Jones.

We know not whether most to admire the diligence of Mr. Dawson in collecting every information, or his candour in acknowledging the sources of improvement. We have often regretted that the practitioners of London read so little. The few secondary hæmorrhages which occurred, the author supposes were venous, as they were easily subdued by compression. The operation proved successful.

Article 6.—*Biographical Sketch of Spalanzani.*

THE account of this industrious experimenter is short, but interesting, inasmuch as it refers to a man whose numerous experiments could not but increase the field of natural knowledge. Yet Spalanzani himself had certainly not that talent of induction, which by teaching him how to institute and conduct his experiments, would have enabled him to draw just and practical inferences. He was moreover, too fond of wonders for an experimental philosopher.

Article 7.—*History of Hydrophobia, with the Appearances on Dissection.* By M. P. C. GORCY, Member of the Legion of Honour, formerly Chief Physician to the Army, and Physician of the Hospital, at Metz.

THIS case, extracted from the *Journal de Medicine, &c.* of Corvissart, Leroux, and Boyer, is in many respects interesting. It terminated like most others. Some circumstances induce the relator to believe that the disease did not arise from the bite of a rabid animal, but was spontaneous Hydrophobia.

The arguments are thus summed up by the compilers.

"1. The dog was young, quarrelled with its fellow, which it

 bit bit severely; and it was in attempting to separate them, that first the huntsman and then the master were bitten.

"2 It was not at the time supposed to be mad, and, during his whole illness, its master never imagined that the bite was the cause.

"3. Neither the other dog, huntsman, nor another child, whom the same dog had bitten, suffered any bad consequences from the bite.

"4. The supposition that the bite of an animal merely enraged can produce hydrophobia is without proof.

"5. Communicated hydrophobia proves commonly fatal on the fourth day; the fatal case of spontaneous hydrophobia, mentioned by Salius Diversus, terminated on the eighth, the duration of the illness in this case.*

"6. If it be correct that the breaking out of the cicatrix, or at least, that swelling and pain in the place bitten, are constant precursors in hydrophobia, these were wanting in this instance."

*Articles* 8, 9, *and* 10, *will be given in our next Publication.*

*A Practical Inquiry into Disordered Respiration, distinguishing the Species of Convulsive Asthma, &c.* By ROBERT BREE, M.D. Fellow of the Royal College of Physicians. The Fourth Edition, with additional Practical Observations.

WE noticed this work with approbation in a former Number. The fourth edition proves, that the inquiry into causes of asthma has interested the public in no common degree. It is enlarged with the result of additional experience and observation, which, at the same time, add authority to the principles of the work.

The author is of opinion, that asthma proceeds from various diseases; and that the spasmodic action of the muscles of the thorax may and do take place, for the purpose of removing irritations or injuries from the abdominal, as well as from the thoracic viscera. Amongst the causes, he particularly notices acrimony in the stomach and bowels, from indigested aliment, or vitiated secretions. Causes of asthma are also said to exist in irritations or diseases of the urinary organs, and of the uterus. Many cases are given in support of these causes of asthma, all of which, with the practical observations, are deserving of serious attention. We will give some extracts from the new matter, which this edition furnishes. Respecting tonics, Dr. Bree remarks, that

"The debility of the vascular system is particularly productive of

"* A case of hydrophobia occurred in Edinburgh last summer, in which the patient lived fifteen days after the first appearance of the disease, and eight months after being bitten. There was no doubt of the disease being communicated, for the dog was destroyed as being mad, and the first symptom was an inflammation, and papulary eruption on the part bitten. In other respects, it had a considerable resemblance to Mr. Gorcy's case."

of a languid circulation in the lungs and liver. A feeble and slow circulation in these organs has been sufficiently pointed out as the cause of serious effusion, or of such a turgid state as may occasion difficulties of breathing, forming a paroxysm of asthma, or a disease of a chronic character. This existing state seems to have caused that apprehension of tonics, which has generally prevailed amongst physicians in the treatment of asthma. But the state now described is that of *passive* weakness, and not *active* tone or inflammatory disposition. It requires, therefore, an appropriate treatment, which has not been usually applied."

The author has seen iron efficacious, even in the paroxysm, in some instances, which, however, he does not give as a rule of general practice.

"It has happened, in several instances, after various means intended to mitigate the distress of the fit had failed, that the rubigo ferri, in doses of ten grains every four hours, appeared most clearly to remove the paroxysm. This effect can only, I think, be accounted for, by looking to the inert condition of the stomach and lungs, and to the languid state of the circulation in the thoracic and abdominal viscera. Whatever, in such circumstances, can hasten the passage of the blood through the lungs, and promote a quicker return to the heart from the lower viscera, must be useful in the intention of present relief, as well as of actual cure."

On the disordered state of the stomach, he offers the following remarks:

"To alter the qualities of the acrimonious matters in the first passages, we may employ acids or absorbents. If a bilious state of the stomach and duodenum be clearly marked, not only the acetous acid, but even the mineral acids much diluted, are very proper. No saccharine acids or acescents will answer the purpose in view, in the asthmatic habit; for if it be the alcaline property of the bile that offends the irritable system, through the medium of the digestive organs, we must still abstain from giving the slightest cause for the encrease of dyspepsia. On this account the stronger acids, which at once neutralize bile and excite the secreting surface of the organ to a better action, must on all occasions be preferred. If, on the contrary, acid or acescent matters prevail most in these passages, the consequence of a vitious secretion, or merely of a weak and disordered digestion, then testaceous or cretaceous powders may be more or less mixed with saline or bitter draughts; and in the same cases, volatile and fixed alkalies may be made useful."

When the stomach is oppressed, gentle evacuants are necessary, before means are employed to subdue spasm.

"If a patient be seized with a paroxysm after having eaten improper articles, as vegetables, salted meat, bacon, pastry, soups, or roasted goose, I should think the muscular re-actions might be attributed to these irritating materials, as I have seen the fit, in some instances, at its height, before they could have been removed from from the stomach; and in many more, when they had not passed the bowels. Great inflation and acidity soon added to the irritations.

"If the influence of such causes be well understood, a dose of opium would not be prescribed before the acrimony had been discharged, though, after its removal, this antispasmodic might be useful.

"It may be supposed that a case of this kind would be readily distinguished, but the fact is otherwise. The violence of the asthmatic affection seems to arrest the whole attention of observers, when a simple evacuation of the stomach is sometimes the only effect required to remove it.

"This was the case of Mr. I. S. who had suffered asthma for a long time, and been treated in the usual way. With great vivacity and intelligence, he is fond of society, and in his enjoyment of company he forgets his rules.

"Preparations of iron were eminently serviceable to him, but not so strikingly useful as rhubarb and magnesia, which were afterwards taken every night by the advice of a judicious apothecary. The fit was prevented by this method; and it is even probable that no other means would be necessary to confirm a cure, if he would be more guarded in his diet."

"Lieutenant S. of the royal navy, had been long affected with disordered secretions of bile, and all the symptoms of indigestion and general debility. When he consulted me, the asthmatic attacks came with great violence, and were evidently excited by the occasional state of the first passages in his feeble habit. The use of gentle aperients with stomachics, was followed by that of vitriolic acid and tincture of cascarilla; and afterwards by vitriolated iron, rhubarb, and pulv. aromat. His general habit was strengthened; his sweats left him, his digestion gradually improved, and the fits did not appear during a long period of progressive improvement of general health. In this state he took a violent cold, but he was but little affected in his breathing. His bowels, however, became more lax than usual; and this disorder, after a few days, increased to a purging. I yielded to his wish, and prescribed chalk mixture and opium with rhubarb. The day after he began this medicine, he congratulated himself that the bowel complaint had ceased; but on the second evening, he had a very violent return of asthma. I then prescribed for him a mixture of magnesia and infusion of senna. His lax state of the bowels soon returned, and the fit was suspended after two motions; the diarrhœa only continued a few days, and left him well.

"But when affections of the liver occasion asthma, there is a great difficulty in the management of the stomach. If cordials and a meat diet be limited considerably, for the purpose of avoiding a turgid state of the liver, the spasmodic breathing is likely to be increased. Such is the habit of an asthmatic subject, that it cannot be concluded without experience in his case, that evacuants will will be beneficial, although the irritating cause be so manifest. Every habit seems to have its peculiar character, subordinate to the general principle of the disease; and it is owing to these idiosyncrasies, that opposing qualities of medicine are found to be useful in different cases of the same complaint. Which of the acids, therefore, may be most efficacious, cannot be safely asserted without some trial.

"The acetous acid has been spoken of, as best applied, when the coats of the first passages are irritated by bilious acrimony, which it may serve to neutralize. This condition is very probably common to asthmatics, although it be not immediately detected amongst the possible causes of the disease.

"The mineral acids are not so likely to answer in relieving the fit as the vegetable acid; but a draught, with nitric acid much diluted, has been given with advantage in both states. The vitriolic acid has been proved to be a powerful tonic, taken three times a day in the intermissions. Tincture of cascarilla adds to its efficacy. The habits in which it was most efficacious, were affected with frequent bilious disturbances of the alimentary canal, and much dyspepsia and eruptions of the skin, with general relaxation and weakness. In these circumstances the improvement of health was progressive, and the fit was lost. But the acetous acid cannot be depended upon for relief in the fit; and when dyspepsia, entirely unconnected with bilious acrimony, is predominant, testaceous powder is to be preferred, sometimes combined with magnesia, and small doses of Ipecacohan.

"Asthmatic attacks take place very commonly, when the viscera of the abdomen are turgid; at the same time that the stomach is torpid, and affected with flatulence and acid juices, and the urine is deficient and high-coloured. It is then that natron shews admirable effects, but not very rapidly; it should be given daily with bitters and rhubarb. It may be united occasionally with an aperient of more force; but four grains of rhubarb every night, and six grains of natron twice or three times in the day, produce a change in the secretions of the alimentary canal, which may, in many instances, remove the asthma without other means. The means, however, of confirming the change of habit, ought never to be neglected—by bitter infusions, and preparations of iron, with a strict diet."

Strong purging is frequently hazardous.

"Whoever attempts it, must bear the ill humour of his patient, and excite many paroxysms for the uncertain attainment of ultimate success. Where purging is apparently necessary from the cause of the complaint, I have found it prudent to neutralize acidities, and dissolve viscid secretions; and in the course of this treatment, to discharge the irritating matters in a gentle manner."

"Those habits in which a more vigorous practice can be adopted, offer exceptions to general rules."

Dr. Bree concludes with that respectful attention to the opinion of his brethren, which evinces his liberality; and is, at all times, honourable to science.

